# A Broad Brush, Global Overview of Bacterial Sexuality

**DOI:** 10.1371/journal.pgen.1002255

**Published:** 2011-08-18

**Authors:** Mark Achtman

**Affiliations:** Environmental Research Institute, University College Cork, Cork, Ireland; Universidad de Sevilla, Spain

Bacterial sexuality is confusing, even for experts! I used to be such an expert on one mechanism of sexuality, conjugation, but that was over 30 years ago. At that time, extra-chromosomal elements, so-called plasmids, were known to encode multiple proteins that together enabled cell-to-cell contacts, which were then used to transfer single-stranded DNA from donor to recipient, thus providing the plasmid with a new host. Transfer of the plasmid resulted in concomitant transfer of any genes that it happened to include, such as genes encoding resistance to antibiotics or virulence factors. On unusual and rare occasions, the plasmid integrated into the chromosome, resulting in the conjugative transfer of chromosomal DNA. What was particularly confusing was the plethora of plasmids that encoded genes for conjugation, each apparently different from the other, and the corresponding large variety of differences between mechanisms of conjugation associated with different plasmids. Some plasmids didn't even encode genes for conjugation, but simply hitch-hiked with the help of conjugation proteins expressed by other plasmids, a phenomenon called mobilization.

Fast forward 30 years, and things became even more confusing. We have learned that conjugation doesn't even need plasmids. So-called integrative conjugative elements (ICEs) are capable of conjugation, but unlike plasmids, which are predominantly free in the cytoplasm, ICEs are integrated into the bacterial chromosome(s). The ends of ICEs contain short stretches that can recombine via site-specific recombination, similar to the excision of bacteriophages or transposons. Like plasmids, conjugation transmits the ICE itself, which first excises from the chromosome within the donor and finally integrates into the recipient genome [1]. But occasionally the ICE also transfers chromosomal DNA, which can correspond to a significant proportion of the entire bacterial genome [2]. And bacterial chromosomes can contain still other transmissible elements, including some that can be mobilized by ICEs, such as integrative and mobilizable elements (IMEs) [1].

Conjugation (and other forms of sexuality such as transduction and transformation) can have dramatic evolutionary consequences. The use of methicillin for medical treatment of staphylococcal disease is now endangered due to the repeated selection [3] of independent staphylococcal variants that contain a methicillin resistance gene that probably evolved in non-pathogenic staphylococci [4]. The repeated acquisition of genomic islands (and the parallel loss of others) has resulted in “open” pan-genomes in some bacterial species [5], such as *Escherichia coli*, in which the variable (dispensable) portion of its genome is more than ten times as large as the conserved core genome [6]. Homologous recombination is as frequent as mutation in many microbial taxa [7], potentially facilitating selective sweeps of novel genes or particularly fit combinations of nucleotides throughout a species. Horizontal gene transfer between taxa is thought to be especially frequent between the inhabitants of a common environmental niche, and can blur or even eliminate patterns of phylogenetic descent [8]. But which particular genetic elements are responsible for these inundations with foreign genes?

Plasmid-encoded conjugation can be subdivided into three genetic modules. The first, increasingly referred to as MOB, consists of a relaxase that nicks double-stranded, super-coiled DNA at a specific *oriT* site. The relaxase forms a so-called relaxosome complex with the terminal nucleotide of the nicked DNA, a single strand of which is then transferred by conjugation. The biochemical details of this nicking and coupling reaction are becoming clearer [9], more so than for the two other modules. The second module consists of a Type IV secretion system, often abbreviated as T4SS. The T4SS is a protein pore through the cell surface, whose magnificently beautiful, basic structure has recently been elucidated in Gram-negative bacteria, in which it connects the inner and outer membrane through the periplasm [10]. T4SS genes are essential for conjugation, and are often genetically linked to genes encoding a pilus, a protein grappling hook that can bind to other cells, or to surface adhesins [11]. T4SS are also sometimes misused by malicious pathogens to inject proteins and DNA into eukaryotic cells and to secrete them into the environment [11], [12]. Finally, the complexed relaxase plus the single-stranded DNA end are transferred to the T4SS secretion system by the third module, consisting of a coupling protein, the T4CP, which links the relaxase-DNA complex to the T4SS and translocates the entire DNA single strand to the recipient. The transferred molecule is then re-ligated by the relaxase. These three modules are associated with a bewildering variety of different gene and protein families in plasmids, whose gene designations are arcane leftovers from the time when I was still an expert in this area. The basis of conjugation by ICEs is more poorly understood, except that the conjugation proteins encoded by some ICEs are quite distinct from those encoded by plasmids [1], [12].

Due to two recent publications from groups led by Eduardo Rocha and Fernando de la Cruz, order is beginning to emerge from chaos, allowing a broad brush overview of the genes that are responsible for conjugation, and of the organisms in which they can be found. In their earlier publication [13], de la Cruz and Rocha examined 1,730 plasmid genomes, half of which were from proteobacteria, and the other half of which were primarily from firmicutes, spirochetes, and actinobacteria. A bioinformatic pipeline assigned most genes encoding relaxases to one of six protein families designated MOB_V_, MOB_Q_, MOB_P_, MOB_H_, MOB_F_, and MOB_C_. Similarly, one protein that is present in almost all T4SS, so-called VirB4/TraU, could be assigned to one of four protein families designated MPF_G_, MPF_T_, MPF_I_, and MPF_F_. T4CPs (also known as VirD4) share homology with VirB4 by BlastP but could be separated into a single T4CP family by the same pipeline. This publication thus provides an initial overview of the number of protein families involved in plasmid conjugation and their associations with phylum. But how about ICEs and their intrachromosomal relatives?

The new publication by Guglielmini et al. in this issue of *PLoS Genetics* [14] addresses this question by scanning the genomes of 1,207 chromosomes and 2,282 plasmids. The pipeline was improved to independently identify relaxases, VirB4, and T4CP on the basis of protein profiles and a hidden Markov model. The assignments with the new pipeline for plasmids resemble those obtained previously. But now it is possible to make quantitative comparisons of the frequency and distribution of conjugation proteins between plasmids, ICEs, and related elements. The first simple answer is that ICEs, defined as the presence of relaxases, VirB4, and T4CP in close proximity, were twice as frequent as were conjugative plasmids, 335 versus 180. An even higher number of chromosomal relaxases (402) were found that were not associated with T4SS, which might reflect the existence of IMEs. The second simple answer is that ICEs were found in proteobacteria, bacteroidetes, firmicutes, cyanobacteria, acidobacteria, fusobacteria, and even in chlorobi. ICEs were found in >50% of genomes from bacteroidetes and some clades of proteobacteria. Only one ICE and one conjugative plasmid were found in archaea, but multiple VirB4 were found, often associated with a T4CP, which suggests that they might be linked to relaxases that were not recognized by the pipeline. Similarly, the number of ICE in actinobacteria was low, but relaxases were common, suggesting that some families of T4SS and T4CP may not have been recognized by the protein profiles. Taken together, these results show that the potential for conjugation is common throughout bacteria and possibly in archaea as well.

These results have several implications. Firstly, plasmids and ICEs share the same protein families, and should be considered as alternative vehicles for conjugation pathways, rather than as distinct entities. Secondly, the potential for horizontal gene transfer and homologous recombination is widespread throughout microbes, which can help explain why mobile genetic elements are so common in their genomes. Finally, conjugative elements provide a ubiquitous mechanism for the facile transmission of genes between discrete clades, whose predominance has not previously been adequately appreciated.

## References

[1] Wozniak RA, Waldor MK (2010). Integrative and conjugative elements: mosaic mobile genetic elements enabling dynamic lateral gene flow.. Nat Rev Microbiol.

[2] Brochet M, Rusniok C, Couve E, Dramsi S, Poyart C (2008). Shaping a bacterial genome by large chromosomal replacements, the evolutionary history of *Streptococcus agalactiae*.. Proc Natl Acad Sci U S A.

[3] Nübel U, Roumagnac P, Feldkamp M, Song JH, Ko KS (2008). Frequent emergence and limited geographic dispersal of methicillin-resistant *Staphylococcus aureus*.. Proc Natl Acad Sci U S A.

[4] Tsubakishita S, Kuwahara-Arai K, Sasaki T, Hiramatsu K (2010). The origin and molecular evolution of the determinant of methicillin-resistance in *Staphylococci*.. Antimicrob Agents Chemother.

[5] Tettelin H, Riley D, Cattuto C, Medini D (2008). Comparative genomics: the bacterial pan-genome.. Curr Opin Microbiol.

[6] Chaudhuri RR, Sebaihia M, Hobman JL, Webber MA, Leyton DL (2010). Complete genome sequence and comparative metabolic profiling of the prototypical enteroaggregative *Escherichia coli* strain 042.. PLoS ONE.

[7] Fraser C, Hanage WP, Spratt BG (2007). Recombination and the nature of bacterial speciation.. Science.

[8] Kloesges T, Popa O, Martin W, Dagan T (2011). Networks of gene sharing among 329 proteobacterial genomes reveal differences in lateral gene transfer frequency at different phylogenetic depths.. Mol Biol Evol.

[9] de la Cruz F, Frost LS, Meyer RJ, Zechner EL (2010). Conjugative DNA metabolism in Gram-negative bacteria.. FEMS Microbiol Rev.

[10] Fronzes R, Schafer E, Wang L, Saibil HR, Orlova EV (2009). Structure of a type IV secretion system core complex.. Science.

[11] Alvarez-Martinez CE, Christie PJ (2009). Biological diversity of prokaryotic type IV secretion systems.. Microbiol Mol Biol Rev.

[12] Juhas M, Crook DW, Dimopoulou ID, Lunter G, Harding RM (2007). Novel type IV secretion system involved in propagation of genomic islands.. J Bacteriol.

[13] Smillie C, Garcillan-Barcia MP, Francia MV, Rocha EP, de la Cruz F (2010). Mobility of plasmids.. Microbiol Mol Biol Rev.

[14] Guglielmini J, Quintais L, Garcillan-Barcia MP, de la Cruz F, Rocha EP (2011). The repertoire of ICE in prokaryotes underscores the unity, diversity and ubiquity of conjugation.. PLoS Genet.

